# Supplementary material for: Molecular encoding and synaptic decoding of context during salt chemotaxis in C. elegans
Source: Nat Commun. 2022 May 27;13:2928. doi: 10.1038/s41467-022-30279-7 (PMC9142520; doi:10.1038/s41467-022-30279-7)
Supplement: Supplementary file 1 — Supplementary Information [file 41467_2022_30279_MOESM1_ESM.pdf]

Supplementary Information.

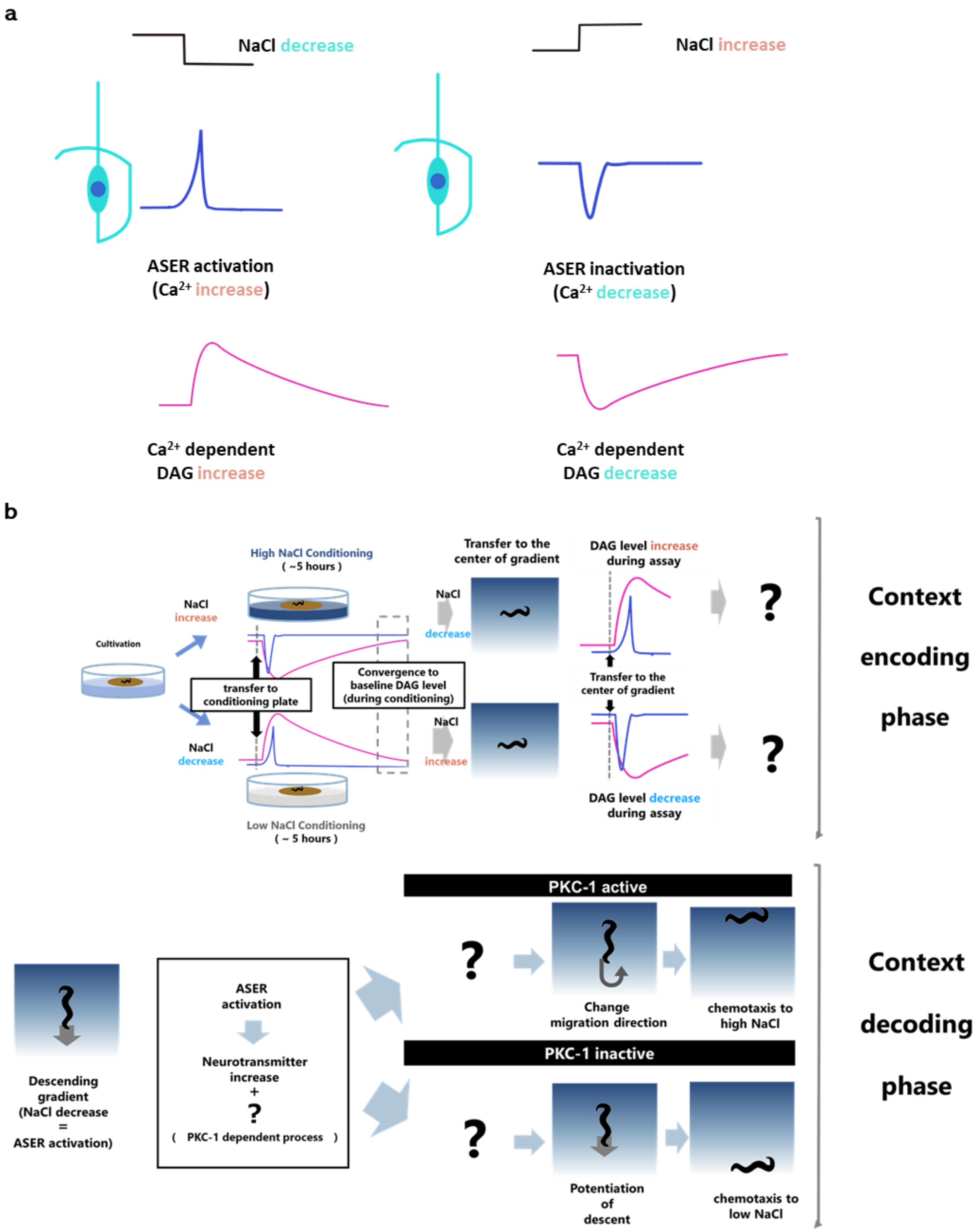

Fig. S1

Schematic illustration of DAG-mediated navigation toward experienced NaCl concentration (Ohno et al., 2017).

(a) Summary of DAG production in ASER[9]. ASER is activated (Ca^{2+} increase) by a decrease in NaCl and deactivated (Ca^{2+} decrease) by an increase in NaCl. Ca^{2+} enhances DAG production (most likely via PLC-beta/EGL-8), and changes in DAG are sustained for longer periods of time (>10 min) compared to changes in Ca^{2+}.(b) DAG-induced behavioral regulation. In the encoding phase (top), when animals are transferred from growth plates to conditioning plates, the amount of DAG in ASER either increases or decreases, according to the difference in the concentration of NaCl between the growth plate and the conditioning plate. However, after ample conditioning time, the amount of DAG returns to baseline. When worms are transferred from the conditioning plate to the center of the NaCl gradient, DAG either increases or decreases depending on the difference between the NaCl concentration at the conditioning plate and the center of gradient plate. Increased or decreased DAG regulates the activity of PKC-1, and PKC-1 signals to unknown molecules to regulate unknown neural processes (‘encoding phase’).

In the decoding phase (bottom), when animals move down the gradient, ASER is activated and neurotransmitter release from ASER increases. This results in bidirectional behavior according to the

molecular state encoded by PKC-1. When PKC-1 is active, worms perform ‘reversal’ behavior to change their migration direction. When PKC-1 is inactive, the reversal behavior is suppressed.

Thus, PKC-1 determines the direction of migration on NaCl gradient.

a

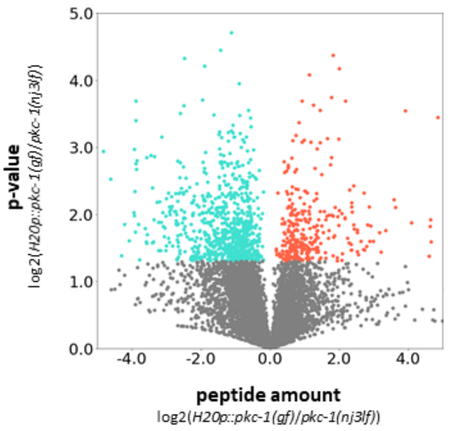

b

No enrichment	Phosphorylation
upregulated phosphopeptide in PKC-1(gf) (P < 0.05)	313
downregulated phosphopeptide in PKC-1(gf) (P < 0.05)	842
Total phosphopeptide identified	8129
Fraction of peptide from Neuron-enriched genes	3.83 %

Fig. S2

Details of phosphoproteomic analysis.

(a) Phosphoproteomic analysis using whole-body extraction from a pan-neuronal *pkc-1(gf)*-expressing strain and *pkc-1(nj3)* mutants. P values were calculated using two-sided t-tests. (b) Summary of phosphoproteomic analyses using whole-body extraction.

Source data are provided as a Source Data file.

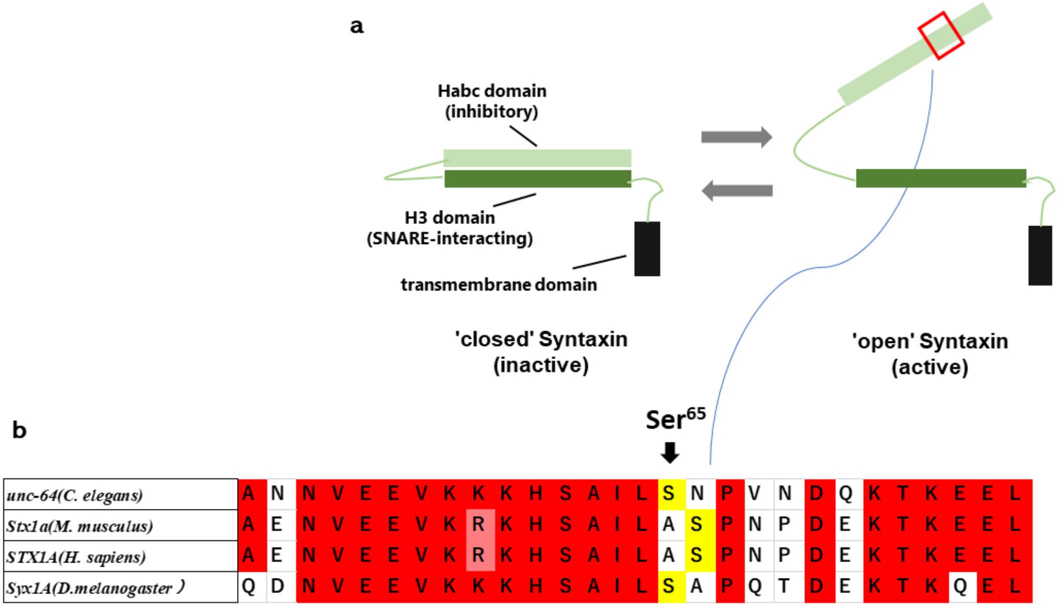

c

Phosphorylation at Ser^{65} homologous site

Species	Evidence
H. sapience	Shiromizu T, et al. (2013), Herskowitz JH, et al. (2010), Xia Q, et al. (2008)
Mus musculus	Trinidad JC, et al. (2012), Huttlin EL, et al. (2010), Wiśniewski JR, et al. (2010), Tweedie-Cullen RY, Reck JM, Mansuy IM (2009)

Fig. S3

Details of UNC-64 Ser65.

(a) The location of Ser65 in UNC-64/Syntaxin 1A. Ser65 is in the autoinhibitory domain, Habc. (b) Alignment of amino acid sequences around UNC-64 Ser65 among multiple species. (c) Several studies have reported phosphorylation at Ser65-homologous sites in mammals.

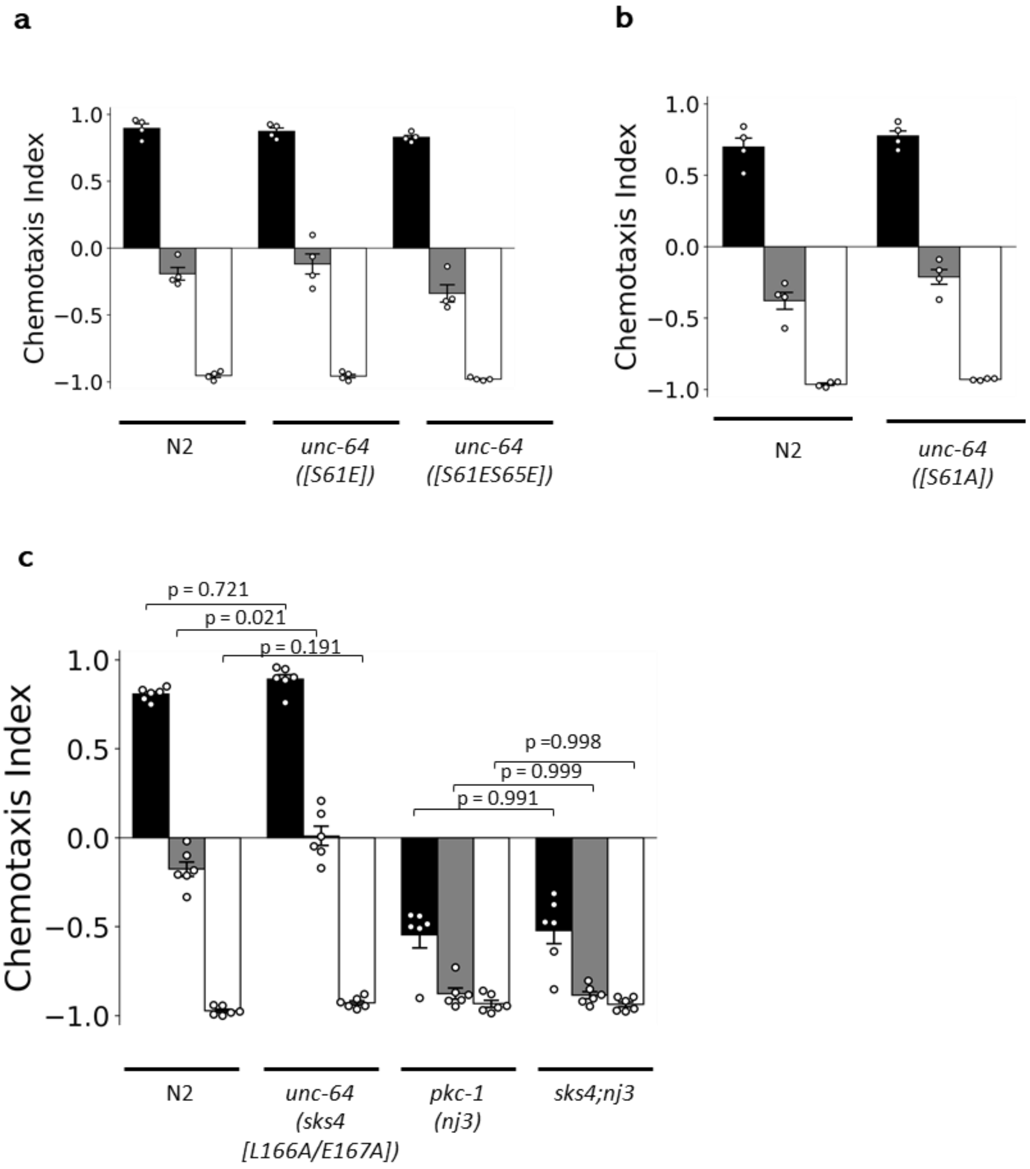

Fig. S4

The analysis of *unc-64* mutants..

(a) Chemotaxis of *unc-64(S to E substitution)* mutants. n = 4 independent experimental repeats(assays)..

(b) Chemotaxis of *unc-64 (S to A at Ser61, the serine adjacent to Ser65)* mutants. The phosphopeptide shown in Fig. 2G also represents the phosphorylation of this residue. n = 4 independent experimental repeats(assays). (c) Chemotaxis of open syntaxin (*unc-64(sks4 L166A/E167A)*) mutants. n = 6 independent experimental repeats(assays). p value were calculated by Tukey test. In Figure S4, Error bars indicate SEM.

Source data are provided as a Source Data file.

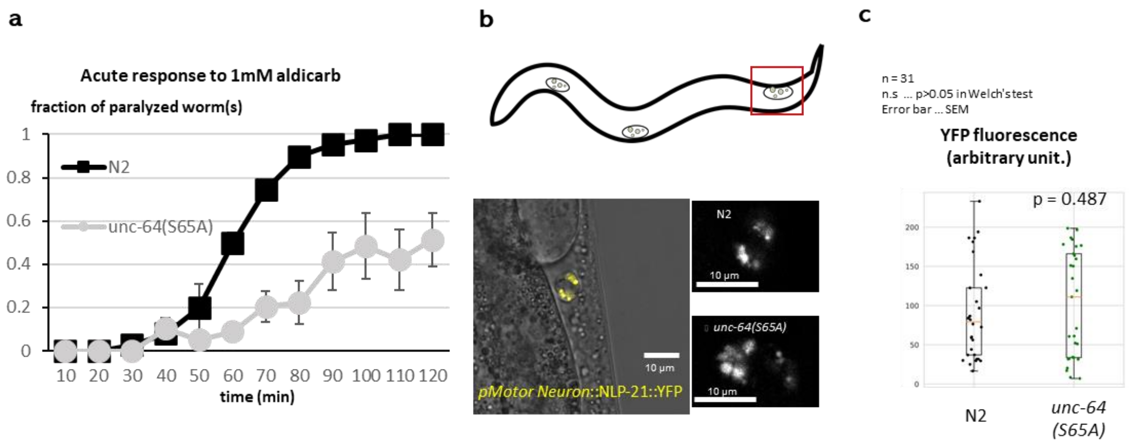

Fig. S5

The function of UNC-64 Ser65 in motor neurons.

(a) Acute paralysis response of N2 and *unc-64(S65A)* strains to 1 mM aldicarb. *unc-64(S65A)* exhibited reduced sensitivity to aldicarb, which suggests *unc-64(S65A)* causes reduced cholinergic synaptic transmission from motor neurons. The error bars show SEM. (b) Coelomocyte uptake assays. Three to four fluorescent puncta (see bottom) were quantified in the posterior coelomocytes (shown in red box). Scale bar: 10 µm. (c) Coelomocyte uptake assay in *unc-64(S65A)* mutants. *unc-64(S65A)* did not exhibit a significant decrease or increase in coelomocyte fluorescence, suggesting that unc-64 Ser65 does not contribute to neuropeptide release. n= 31 animals. n.s.: $p > 0.05$ in Welch's test. (D) The locomotion rate (=body bends) of *unc-64(S65A)* mutants. As predicted from panel A, *unc-64(S65A)* mutants show a defect in locomotion. n = 18(N2), 17 (*unc-64(S65A)*) animals. Error bars indicate SEM. p value were

calculated by Welch's test. The boxes extends from 25th to 75th percentile, median is marked by the line, and whiskers indicate the minimum and the maximum values in 1.5x the inter-quartile range.

Source data are provided as a Source Data file.

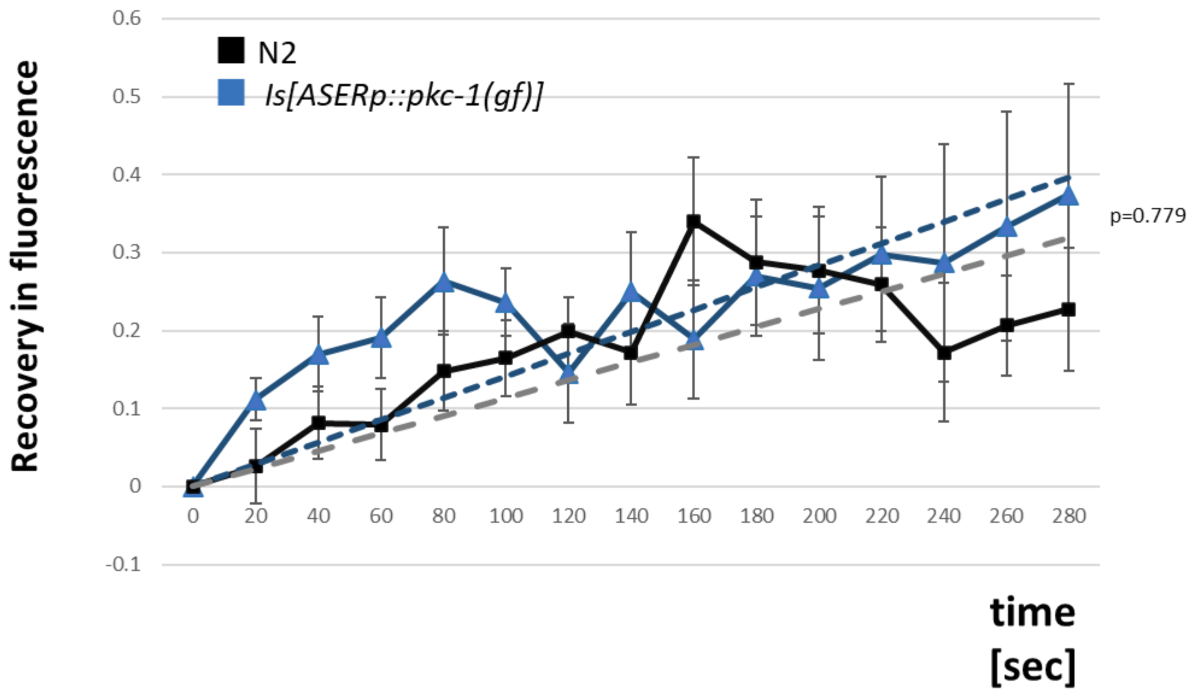

Fig. S6

ASERp::*pkc-1*(gf) does not significantly enhance baseline glutamate release.

FRAP experiments in *Is[ASERp::pkc-1(gf)]*. p value were calculated by t-value of interaction between time course and strains in generalized linear model). Error bars indicate SEM. n = 8(pkc-1(gf), 9(N2) independent animals, respectively. Source data are provided as a Source Data file.

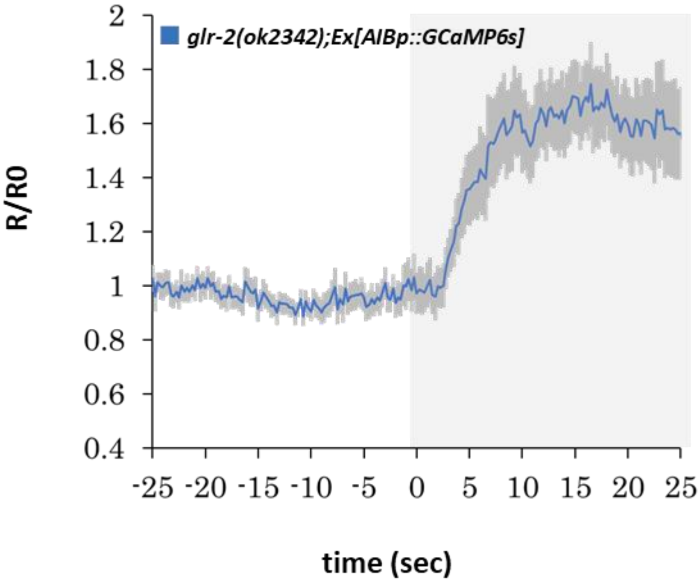

Fig. S7.

***glr-2*, another glutamate receptor gene expressed in AIB, does not contribute to the AIB response to NaCl.**

The response of AIB in *glr-2(ok2342)* to NaCl concentration change from 50 mM to 25 mM. *glr-2(ok2342)* showed obvious excitatory responses, suggesting that *glr-2* is not required for receiving the glutamate transmission from ASER. n = 21 animals. The imaging setup and following analysis were exactly the same as Sato et al., 2021[30]. Source data are provided as a Source Data file.

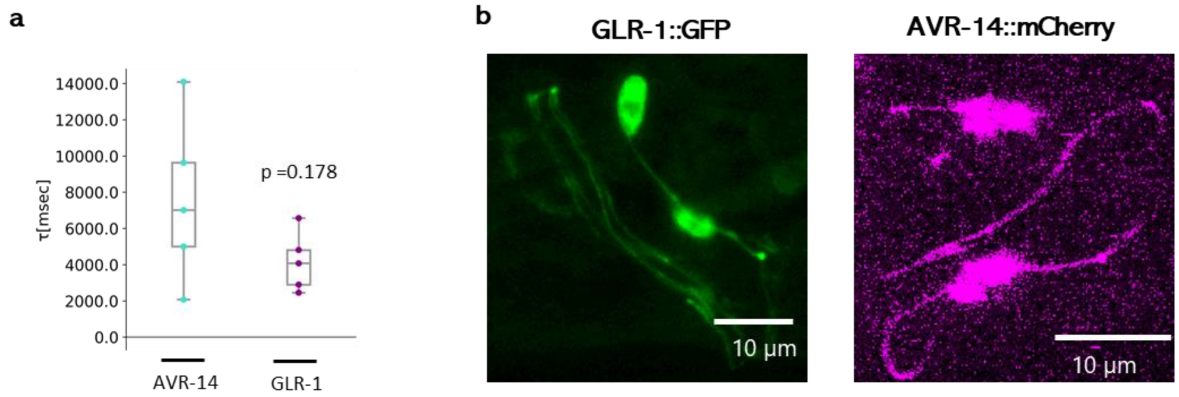

Fig.S8.

The features of glutamate receptors, AVR-14 and GLR-1.

(a) desensitization time constant of AVR-14 and GLR-1. The AVR-14 dataset is from Fig.7, except one sample which was omitted because of the short time of glutamate application. The GLR-1 dataset is newly acquired, because in the dataset of Fig. 7, oocytes were clamped longer and the waveform was somewhat noisy. n = 4 oocytes. p value were calculated by Welch's test. The boxes extends from 25th to 75th percentile, median is marked by the line, and whiskers indicate the minimum and the maximum values in 1.5x the inter-quartile range. (b) Rough localization patterns of AVR-14 and GLR-1. The receptors did not appear to localize at specific sites in the axon. Scale bar: 10 µm.

Source data are provided as a Source Data file.
