# Supplementary material for: Molecular encoding and synaptic decoding of context during salt chemotaxis in C. elegans
Source: Nat Commun. 2022 May 27;13:2928. doi: 10.1038/s41467-022-30279-7 (PMC9142520; doi:10.1038/s41467-022-30279-7)
Supplement: Supplementary file 3 — Description of Additional Supplementary Files [file 41467_2022_30279_MOESM3_ESM.pdf]

Description of Additional Supplementary Files:

File Name:

Supplemental Data S1_PKC-1_TurboID_phosphoproteome

Description: The summary data of phosphoproteomic analysis using TurboID in Fig.2

File Name:

Supplemental Data S2_PKC-1_TurboID_total_proteome.xlsx

Description:

The summary data of proteomic(non-phospho-enriched) analysis using TurboID.
