# Supplementary material for: Molecular encoding and synaptic decoding of context during salt chemotaxis in C. elegans
Source: Nat Commun. 2022 May 27;13:2928. doi: 10.1038/s41467-022-30279-7 (PMC9142520; doi:10.1038/s41467-022-30279-7)
Supplement: Supplementary file 6 — Reporting Summary [file 41467_2022_30279_MOESM6_ESM.pdf]

nature portfolio

Corresponding author(s): Yuichi Iino

Last updated by author(s): Apr 7, 2022

Reporting Summary

Nature Portfolio wishes to improve the reproducibility of the work that we publish. This form provides structure for consistency and transparency in reporting. For further information on Nature Portfolio policies, see our Editorial Policies and the Editorial Policy Checklist.

Statistics

For all statistical analyses, confirm that the following items are present in the figure legend, table legend, main text, or Methods section.

n/a	Confirmed	
☐	☒	The exact sample size (*n*) for each experimental group/condition, given as a discrete number and unit of measurement
☐	☒	A statement on whether measurements were taken from distinct samples or whether the same sample was measured repeatedly
☐	☒	The statistical test(s) used AND whether they are one- or two-sided<br>*Only common tests should be described solely by name; describe more complex techniques in the Methods section.*
☐	☒	A description of all covariates tested
☐	☒	A description of any assumptions or corrections, such as tests of normality and adjustment for multiple comparisons
☐	☒	A full description of the statistical parameters including central tendency (e.g. means) or other basic estimates (e.g. regression coefficient) AND variation (e.g. standard deviation) or associated estimates of uncertainty (e.g. confidence intervals)
☐	☒	For null hypothesis testing, the test statistic (e.g. *F*, *t*, *r*) with confidence intervals, effect sizes, degrees of freedom and *P* value noted<br>*Give P values as exact values whenever suitable.*
☒	☐	For Bayesian analysis, information on the choice of priors and Markov chain Monte Carlo settings
☒	☐	For hierarchical and complex designs, identification of the appropriate level for tests and full reporting of outcomes
☒	☐	Estimates of effect sizes (e.g. Cohen's *d*, Pearson's *r*), indicating how they were calculated

Our web collection on statistics for biologists contains articles on many of the points above.

Software and code

Policy information about availability of computer code

Data collection	We used Metamorph(version7.7.3) to acquire image of fluorescent probes, pClamp8.2 to acquire electrophysiological data, LAS-AF to acquire confocal images of fluorescent proteins.
Data analysis	We used Fiji(1.52p),Metamorph(version7.7.3), Clampfit(10.3.2.1), Proteome Discoverer 2.2, R(RStudio,1.2. 5019), Python(3.7.13), Microsoft Excel(Microsoft 365)

For manuscripts utilizing custom algorithms or software that are central to the research but not yet described in published literature, software must be made available to editors and reviewers. We strongly encourage code deposition in a community repository (e.g. GitHub). See the Nature Portfolio guidelines for submitting code & software for further information.

Data

Policy information about availability of data

All manuscripts must include a data availability statement. This statement should provide the following information, where applicable:

- Accession codes, unique identifiers, or web links for publicly available datasets
- A description of any restrictions on data availability
- For clinical datasets or third party data, please ensure that the statement adheres to our policy

The mass spectrometry proteomics data have been deposited to the ProteomeXchange Consortium via the PRIDE partner repository with the dataset identifier PXD031536.
The summary of the analysis was provided as Supplemental S1 and S2.

Field-specific reporting

Please select the one below that is the best fit for your research. If you are not sure, read the appropriate sections before making your selection.

☒ Life sciences ☐ Behavioural & social sciences ☐ Ecological, evolutionary & environmental sciences

For a reference copy of the document with all sections, see nature.com/documents/nr-reporting-summary-flat.pdf

Life sciences study design

All studies must disclose on these points even when the disclosure is negative.

Sample size	We did not perform sample size test. We determined sample sizes based on related studies: Behavioral tests: Kunitomo et al., 2013, Tsunozaki et al., 2007, Imaging: Sato et al., 2021, Ventimiglia et al., 2017, Samuel et al., 2003, Sieburth et al., 2006.
Data exclusions	Images with no detectable signal, severe failure in tracking of animals, or severe z-axis displacement of specimen was discarded.
Replication	The number of replications are included in figure legends. All replications were performed with appropriate controls.
Randomization	For each experiments, experimental groups were based on genotypes.<br>For individual assay, worms were randomly selected from multiple growth plates.<br>For population assays, animals were allocated to different conditioning plates. We collected all the hundreds of worms from growth plates, washed them with buffer, and placed adequate amount of buffer(typically with ~200 worms) onto the conditioning plates with each concentration of NaCl. Therefore, animals were randomly allocated to each conditions.<br>Strains were assayed in parallel or in alternated order. .
Blinding	For behavioral assays with mutant strains, the experimenter was not blinded. Since mutant animals mostly have some phenotypic features(small, slow, fat, etc.), the complete blind test was impossible. For behavioral assays with transgenic animals, the researcher is blinded to Tg(+), since we used fluorescent markers. For imaging experiments, most of processes were performed by semi-automated software, therefore the role of the experimenter is minimal.

Reporting for specific materials, systems and methods

We require information from authors about some types of materials, experimental systems and methods used in many studies. Here, indicate whether each material, system or method listed is relevant to your study. If you are not sure if a list item applies to your research, read the appropriate section before selecting a response.

Materials & experimental systems

n/a	Involved in the study	
☒	☐	Antibodies
☒	☐	Eukaryotic cell lines
☒	☐	Palaeontology and archaeology
☐	☒	Animals and other organisms
☒	☐	Human research participants
☐	☐	Clinical data
☒	☐	Dual use research of concern

Methods

n/a	Involved in the study	
☒	☐	ChIP-seq
☒	☐	Flow cytometry
☒	☐	MRI-based neuroimaging

Animals and other organisms

Policy information about studies involving animals; ARRIVE guidelines recommended for reporting animal research

Laboratory animals	Caenorhabditis elegans (Adult day 1-2 animals.) hermaphrodite.<br>Strains used are :BristolN2,pkc-1(nj3),pkc-1(nj3);Ex[gcy-5p::pkc-1],pkc-1(nj3);Ex[gcy-5p::pkc-1(A160Egf)],Is[H20p::pkc-1(gf);unc-122p::mcherry],Is[rgef-1p::TurboID;myo-3p::venus];Is[H20p::pkc-1(gf);unc-122p::mcherry],nj3;Is[rgef-1p::TurboID;myo-3p::venus],unc-64([Ser65Ala]),Si[H20p::frt::unc-64(WT)::frt::let-858 3'UTR::GFP::unc-54 3'UTR;Cb unc-119];unc-64(pe[Ser65Ala]),Ex[gcy-5p::nFLP;unc-122p::mcherry],Ex[gcy-5p::eat-4::pHluorin;lin-44p::mcherry],pkc-1(nj3);Is[gcy-5p::eat-4::pHluorin;lin-44p::mcherry],Is[gcy-5p::eat-4::pHluorin;lin-44p::mcherry],unc-64(S65A);Is[gcy-5p::eat-4::pHluorin;lin-44p::mcherry],eat-4(ky5);Is[gcy-5p::pkc-1(gf)],unc-31(e928);Is[gcy-5p::pkc-1(gf)],ky5;e928;Is[gcy-5p::pkc-1(gf)],kySi76kySi77[frt::eat-4genomic CDS::frt::let-858 UTR::mcherry::eat-4 genomic 3'UTR];Is[gcy-5p::nFLP;unc-122p::venus],unc-64(S65A);kySi76kySi77[frt::eat-4 genomic CDS::frt::let-858 UTR::mcherry::eat-4 genomic 3'UTR];peIs[gcy-5p::nFLP;unc-122p::venus],Ex[npr-9p::InversePericam216a;npr-9p::mcherry;lin-44p::venus],unc-64(S65A);Ex[npr-9p::InversePericam216a;npr-9p::mcherry;lin-44p::venus],pkc-1(nj3);Ex[npr-9p::InversePericam216a;npr-9p::mcherry;lin-44p::venus],avr-14(ad1302);pkc-1(nj3);Ex[npr-9p::InversePericam216a;npr-9p::mcherry;lin-44p::venus],pkc-1(nj3);Ex[gcy-5p::pkc-1(gf)];Ex[npr-9p::GCaMP6s;npr-9p::mcherry;lin-44p::venus],glr-1(ky176);pkc-1(nj3);Ex[gcy-5p::pkc-1(gf)];Ex[npr-9p::GCaMP6s;npr-9p::mcherry;lin-44p::

venus],unc-13(e51);Ex[npr-9p::InversePericam216a;lin-44p::venus],unc-13(e51);Ex[npr-9p::GCaMP6s;npr-9p::mcherry;lin-44p::venus],avr-14(ad1302)unc-13(e51);Ex[npr-9p::InversePericam216a;lin-44p::venus],unc-13(e51);glr-1(ky176);Ex[npr-9p::GCaMP6s;npr-9p::mcherry;lin-44p::venus],unc-64([Ser61Glu]),unc-64([Ser61GluSer65Glu]),unc-64([Ser61A]),Ex[gcy-5p::GCaMP2.0;lin-44p::GFP],pkc-1(nj3);Ex[gcy-5p::GCaMP2.0;lin-44p::GFP],Ex[gcy-5p::pkc-1(gf)];Ex[gcy-5p::GCaMP2.0;lin-44p::GFP],Is[gcy-5p::eat-4::pHluorin;lin-44p::mcherry];Is[gcy-5p::pkc-1(gf);lin-44p::GFP],Is[npr-9p::glr-1::GFP,lin-44],Ex[npr-9p::avr-14::mcherry]

Wild animals	This study does not include wild animals, but common C. elegans strains(Bristol N2) used broadly was used as wild type.
Field-collected samples	This study does not involve field-collected samples.
Ethics oversight	The invertebrate Caenorhabditis elegans does not require ethical approval or overviews.

Note that full information on the approval of the study protocol must also be provided in the manuscript.

Clinical data

Policy information about clinical studies

All manuscripts should comply with the ICMJE guidelines for publication of clinical research and a completed CONSORT checklist must be included with all submissions.

Clinical trial registration	*Provide the trial registration number from ClinicalTrials.gov or an equivalent agency.*
Study protocol	*Note where the full trial protocol can be accessed OR if not available, explain why.*
Data collection	*Describe the settings and locales of data collection, noting the time periods of recruitment and data collection.*
Outcomes	*Describe how you pre-defined primary and secondary outcome measures and how you assessed these measures.*
